# Recovery in schizophrenia: A network analysis of inter-relationships among disease-related variables, personal resources, context-related factors and real-life functioning

**DOI:** 10.1192/j.eurpsy.2021.144

**Published:** 2021-08-13

**Authors:** A. Mucci, S. Galderisi, P. Rucci, A. Rossi, P. Rocca, A. Bertolino, M. Maj

**Affiliations:** 1 Department Of Psychiatry, Univeristy of Campania Luigi Vanvitelli, Naples, Italy; 2 Department Of Psychiatry, University of Campania "Luigi Vanvitelli", NAPOLI, Italy; 3 Department Of Biomedical And Neuromotor Sciences, University of Bologna, Bologna, Italy; 4 Department Of Biotechnological And Applied Clinical Sciences, Section Of Psychiatry, University of L’Aquila, L’Aquila, Italy; 5 Department Of Neuroscience, University of Turin, Torino, Italy; 6 Department Of Neurological And Psychiatric Sciences, University of Bari, Bari, Italy; 7 Psychiatry, Università degli Studi della Campania “Luigi Vanvitelli”, Napoli, Italy

## Abstract

**Abstract Body:**

Central to recovery-oriented approaches in schizophrenia are treatment integration and personalization, targeting key variables beyond symptom reduction. The Italian network for research on psychoses conducted a study demonstrating, using network analysis, the central role of community activities in bridging the effects of symptoms, cognition, functional capacity and service engagement on real-word functioning. A 4-year follow-up study was recently completed and the presentation will illustrate the findings. Network analysis was used to test whether relationships among all variables at baseline were similar at follow-up. In addition, the network structure was compared between subjects classified as recovered or non-recovered at follow-up. Six hundred eighteen subjects were assessed at both baseline and 4-year follow-up. Results showed that the network structure was stable from baseline to follow-up, and the overall strength of the connections among variables did not significantly change. Functional capacity and everyday life skills were the most central variables in the network at both baseline and follow-up, while psychopathological variables were more peripheral. The network structure of non-recovered patients was similar to the one observed in the whole sample, but very different from that of recovered subjects, showing few connections among the different nodes. These data strongly suggest that connections among symptoms/dysfunctions tend to maintain over time, contributing to poor outcome in schizophrenia. Early treatment plans, targeting variables with high centrality, might prevent the emergence of self-reinforcing networks of symptoms and dysfunctions in people with schizophrenia.

**Disclosure:**

Armida Mucci has been a consultant and/or advisor to or has received honoraria from Gedeon Richter Bulgaria, Janssen Pharmaceuticals, Lundbeck, Otsuka, Pfizer and Pierre Fabre. None of these has any impact on this abstract and on the presented study.

